# The Value-Added Contribution of Exercise Commitment to College Students’ Exercise Behavior: Application of Extended Model of Theory of Planned Behavior

**DOI:** 10.3389/fpsyg.2022.869997

**Published:** 2022-05-26

**Authors:** Wen-Juan Zhang, Menglin Xu, Yu-Juan Feng, Zhi-Xiong Mao, Zeng-Yin Yan, Teng-Fei Fan

**Affiliations:** ^1^Department of Physical Education, Qufu Normal University, Qufu, China; ^2^Department of Internal Medicine, Columbus, OH, United States; ^3^Teaching Department of Common Courses, Shandong University of Art and Design, Jinan, China; ^4^School of Psychology, Beijing Sport University, Beijing, China; ^5^School of Physical Education, Chongqing University of Posts and Telecommunications, Chongqing, China; ^6^School of Sports Science, Qufu Normal University, Qufu, China

**Keywords:** college students, exercise commitment, physical exercise behaviors, theory of planned behavior, exercise intention

## Abstract

The aim of this study was to investigate the applicability of the planned behavior theory model (TPB-5) and TPB-6 model of enhanced physical exercise in college students, and to explore the role of exercise commitment in the relationship between exercise intention and behavior, so as to provide theoretical and empirical support for college students to promotion exercise. The study participants were 581 college students (male = 243, female = 338, age = 19.27 ± 0.94) are investigated with Theory of Planned Behavior (TPB) Scale, Exercise Commitment Scale, and Physical Activity Rating Scale. Results showed that the explanatory power of the TPB to exercise intention and exercise behavior is 0.70 and 0.52, respectively, and exercise intention was the primary factor to predict exercise behavior of college students. The Model fit of TPB-6 model is acceptable, compared with TPB 5-factor model, the predictive power of TPB-6 (with the mediator: exercise commitment) on behavioral intention increases from 70.0 to 75.0%, and the predictive power towards behavior raises from 52.0 to 59.0%. Exercise commitment has a partial mediating effect between exercise intention and behavior, which accounts for 26.89% of the total effect, but it has no moderating effect. In conclusion, this research demonstrates the TPB-5 model has good applicability among the college students, with exercise commitment variables, exercise intention can better predict college students’ exercise behavior, which can be used as the theoretical basis for the intervention on their exercise behavior.

## Introduction

The proposition that participation in short- and long-term physical exercise has both physical and psychological benefits is widely known and supported by previous research. Currently, physical inactivity has become the highest risk factor for death from non-communicable diseases worldwide (Bull et al., 2020; Zhu et al., 2020). College students are reserve forces of the society, and the proportion of them who regularly participate in physical exercise are less than 30% (Dong and Zhang, 2016; Yang et al., 2020). Among these students, the obesity and myopia rates remain high, and their cardiopulmonary function does not reach the appropriate level at that age (Van Sluijs et al., 2021; Zhang et al., 2021). Moreover, self-inflicted injuries caused by mental and psychological disorders are frequent, and the health condition of college students is of great concern (Qiu et al., 2011; Yu et al., 2021). Therefore, it has been a common concern of universities and society to promote college students to actively participate in physical exercise, avoid being sedentary and build a healthy body. And it has also become an issue of scientific research to explore the psychological mechanism for the promotion of exercise behavior of college students and construct a predictive intervention model suitable for this group, so as to enable them to keep exercising regularly (Rosenkranz et al., 2021; Liao et al., 2022).

In the area of physical exercise intervention, researchers have constructed multiple theoretical models to provide a strong theoretical basis for physical exercise intervention, among which the most widely-used and mature one is Theory of Planned Behavior (TPB) proposed by Ajzen (1991). the core proposition of TPB is that behavioral intention and perceived behavioral control are the strongest predictors for behavior, while individuals’ attitudes towards behavior, subjective norms and perceived behavioral control, in turn, can predict their behavioral intentions (Plotnikoff et al., 2012; Qiu and Yang, 2017). TPB has contributed to the prediction and intervention of physical exercise, but it also has some shortcomings, that is, the predictive power of the model for behavioral intentions is far higher than that for behavior itself. The “high intention and low behavior” structure indicates that there is a “gap” between intention and behavior (Sheeran, 2002; Gomes et al., 2018; Kang et al., 2020). In order to bridge this “gap” and improve the predictive power of the TPB model for physical exercise behavior, scholars have further improved the TPB model by introducing new variables, integrating the models and adjusting the structural relationships, which has achieved positive effect (Zhang and Mao, 2016; Yang et al., 2020; Rhodes et al., 2021; Arigo et al., 2022; Liao et al., 2022).

The main purpose of bridging the “gap” between intention and behavior is to improve the predictive power of the original TPB model. In the case of college students, exercise commitment plays an important role in the process from behavioral intention to behavioral execution (Dong, 2013). Exercise commitment refers to a kind of positive psychological state that exercisers desire and resolve to continue the physical exercise (Chen and Li, 2007), which is also a driving force to keep exercising adherence. Studies have found that the exercise commitment made by college students before physical exercise can predict their execution of the actual behavior, reflecting the individuals’ determination and loyalty to physical exercise (He, 2011; Sun et al., 2011; Williams, 2013; Weiss, 2021; Hong and Li, 2022). The research made by Dong Wenbo has showed that exercise commitment acts as a partial mediator in the transformation from consciousness to behavior among middle school students, and exercise consciousness can be better transformed into exercise behavior by enhancing the level of exercise commitment (Dong, 2013). The above studies on bridging the gap between intention and behavior are mainly based on the discussion that takes exercise commitment as a mediator, and it acting as a partial mediator between intention and behavior.

Some studies reported that both exercise commitment and intrinsic motivation are incentives for behavior, and also the rational factors that individuals maintain on the basis of cognitive strategies, which makes individuals more inclined to participate in physical exercise (Wu et al., 2010; Qiu et al., 2012; Zhu and Dong, 2016; Jeng et al., 2020). People with strong exercise commitment usually have clear and explicit exercise intentions and goals. On the contrary, if individuals lack exercise commitment (i.e., having weak exercise intentions and determination), some inappropriate behaviors may appear in the face of physical exercise, such as procrastinating and giving up (Dong and Mao, 2020). In the experiment of health behavior intervention, Brinthaupt found that improving exercise commitment had a significant effect on healthy habits and regular exercise (Brinthaupt et al., 2013). Rhodes concluded that there was a higher correlation between exercise intention and actual exercise behavior for individuals with higher exercise commitment and perceived behavioral control (Rhodes and Matheson, 2005). Many studies have shown that exercise commitment is an important factor in the process of exercise behavior that prompts the transformation from behavioral intention to behavior (Wilson et al., 2004; Gabriele et al., 2011; Dong and Zhang, 2016; Frayeh and Lewis, 2016; Wu, 2016; Robbins et al., 2017). These studies suggest that the level of exercise commitment regulates the relationship between intention and behavior, which acts as a moderator between intention and behavior.

The shortcomings of TPB model lies in the gap between intention and behavior, caused by the failure to consider the potential influence of individual personality factors on their behavior, such as motivation, interests, hobbies, and emotions. From the perspective of psychological attitude, commitment is integrated and formed by individual cognitive, affective, volitional and behavioral factors (Xie, 2016; Bum, 2018) so that it is the psychological variable that bridges the gap between intention and behavior. However, current studies on exercise commitment mainly focus on the validation and restructuring of this theoretical model, and few studies incorporate this variable into a complete theoretical framework of exercise behavior for analysis. Given that, this study implants exercise commitment into TPB model (the 5-factor model of physical exercise) to form the 6-factor model of enhanced physical exercise, aiming to examine the application of exercise commitment to the social cognitive theory framework (TPB) and probe into the mechanism of exercise commitment in the transformation from intention to behavior, thus providing a theoretical guidance for the promotion of exercise participation of college students. Therefore, exercise commitment is a psychological link between exercise intention and behavior, and also a psychological contract to enhance individuals’ exercise behavior, which has an influence on students’ overt exercise behavior. For this reason, much attention should be paid to improve the physical exercise commitment of college students, with the purpose of explaining and predicting their exercise behavior, and then providing a theoretical basis for promoting the exercise behavior intervention.

**Figure 1:**
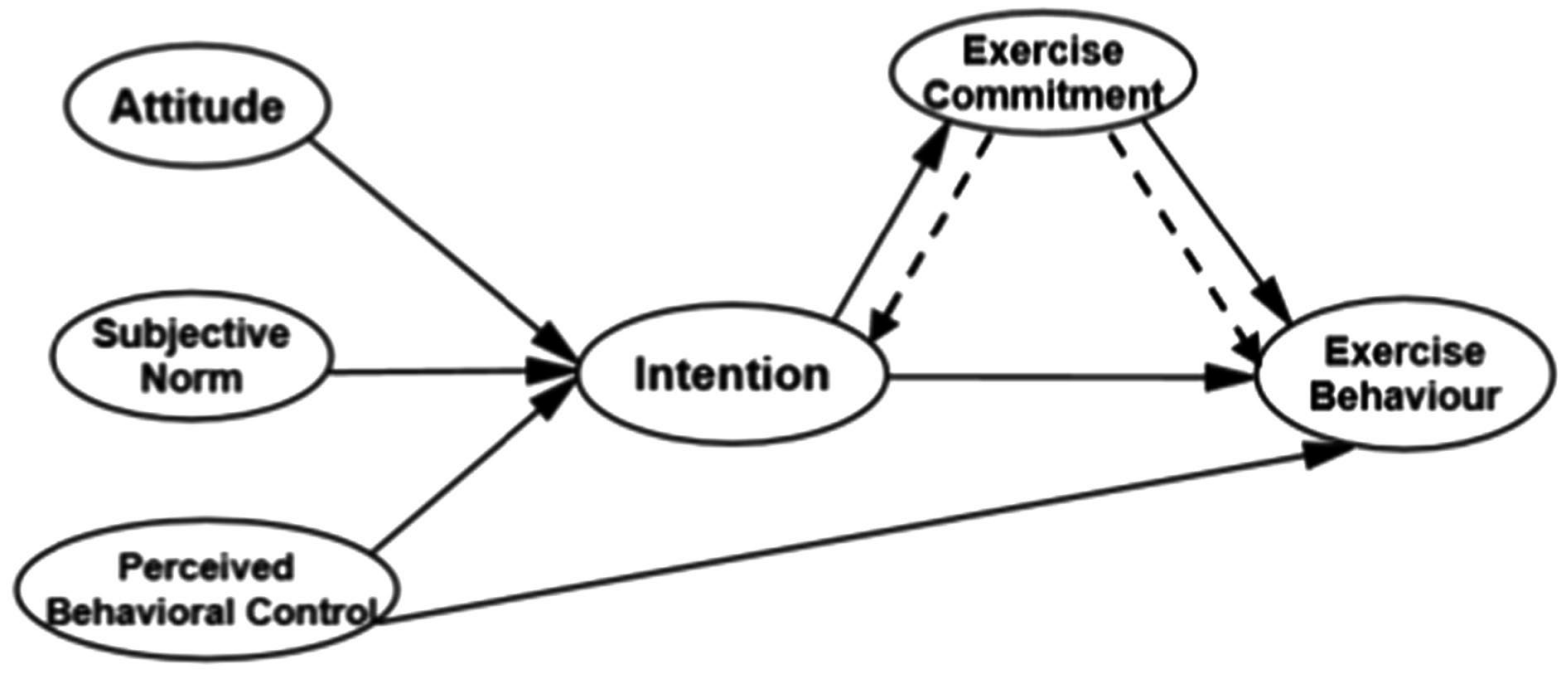
Path coefficient diagram of structural equation model for TPB-6.

In conclusion, this study puts forward the following hypotheses: (1) The TPB can significantly predicts exercise behavior and has good practicability in college student population. (2) Exercise commitment plays a mediating or moderating role between exercise intention and behavior. (3) The 6-factor model of enhanced physical exercise with exercise commitment can better predict exercise behavior of college students (Figure 1). This study aims to clarify the practicability of the theoretical model of planned behavior in college student groups, and to investigate in-depth the role of exercise commitment in the exercise intention–behavior relationship, and then to provide theoretical guidance for promoting college students’ physical exercise participation.

## Method and Participants

### Participants and Procedures

The participants were 610 general undergraduate students in their first and second years from three different types of universities in China (including: Normal education, science and technology, and medicine). After removing those with invalid responses, 581 (95.24%) valid samples were collected. There were 243 males, 338 females, and the mean age was 19.27 (SD = 0.94).

This study obtained the support of university administrative leaders and physical education teachers, after they were introduced to the purpose of this study and filled out anonymously, participants signed an informed consent form, the questionnaire took approximately 15 min to complete, and participants were given a small gift, and this study was carried out in accordance with the ethical guidelines of the American Psychological Association (APA).

### Research Method

#### Assessment Tools

The TPB scale, exercise commitment scale, physical activity level scale-3, and demographic information such as gender, grade, and birthday were included. The details are as follows.

Ajzen’s planned behavior theory includes the subscales of attitudes, subjective norms, perceived behavioral controls, and intentions (Ajzen, 1991). Hu (2008) revised and compiled the TPB scale in China in 2008. This study consists of 14 items rated on a 6-point Likert scale. Ranging from 1 (strongly disagree) to 6 (strongly agree). Example items include: intention “e.g., In the next 4 weeks, I plan to engage in physical exercise at least three times a week for more than 20 min each time,” etc. The internal consistency reliability of the scale ranges from Cronbach’s *a* = 0.754 to 0.893, and its subscales were reported to have decent internal reliability. For each subscale, item scores were averaged to create the composite scores.

The exercise commitment of college students adopts the revised Exercise Commitment Scale by Chen and Li (2007), which consists of 23 items rated on a 5-point Likert scale from 1 (“strongly disagree”) to 5 (“strongly agree”). It includes the subscales of exercise enjoyment, personal investments, social constraints, valuable opportunities, and other priorities. Example items include: “I desire to have enough time and opportunities to participate in physical activity” and “I get a special sense of satisfaction from physical exercise and sport,” etc. The scale has a reliability of Cronbach’s *a* = 0.936. Item scores were averaged to create the composite scores for commitment.

Physical exercise behavior was measured using the physical activity level scale-3 revised by Liang Deqing, which had a retest reliability of 0.82, and an internal consistency coefficient of 0.75 (Liang, 1994). The scale contains three questions on exercise intensity, time and frequency, and uses a 5-level Likert scale. Example items include: “What is the intensity of your physical exercise?” etc. The scale is well-validated by the previous research and is a relatively mature measurement tool, so its reliability is not tested in our study.

#### Statistical Methods

Descriptive statistics of means and standard deviations (SDs) were used to summarize the study variables. Pearson correlation test was used to display the associations among the variables. The software of SPSS 23.0 was used for the data management and correlation test. The potential mediation and moderation effect of exercise commitment (1 model for each) were tested using PROCESS macro. The 5-factor and the extended TPB model were fitted using AMOS 21.0.

## Results

### Descriptive Statistics and Pearson Correlation Among the Variables in the Extended TPB Model

As shown in Table 1, participants had higher mean scores on intentions, attitude, and perceived behavioral control than on exercise behavior and exercise commitment. The correlation test showed that the six variables involved in the extended-TPB model were positively and significantly correlated. The moderate associations among the variables suggested that they were both independent and interconnected, and were well-suited for the SEM analyses.

**Table 1:** Descriptive statistics and Pearson correlation between the variables in the extended TPB model (*n* = 581).

	Variables	1	2	3	4	5	6	*M*	SD
1.	IN	1						4.17	1.21
2.	AT	0.690^**^	1					4.55	1.06
3.	SN	0.473^**^	0.599^**^	1				4.59	1.13
4.	PBC	0.550^**^	0.495^**^	0.368^**^	1			4.15	1.31
5.	EB	0.526^**^	0.455^**^	0.330^**^	0.468^**^	1		3.11	0.81
6.	EC	0.529^**^	0.565^**^	0.406^**^	0.383^**^	0.470^**^	1	3.01	0.75

IN, intentions; AT, attitude; SN, subjective norms; PBC, perceived behavioral control; EB, exercise behavior; EC, exercise commitment.

** *P* < 0.01.

### The 5-Factor TPB Model

**Figure 2:**
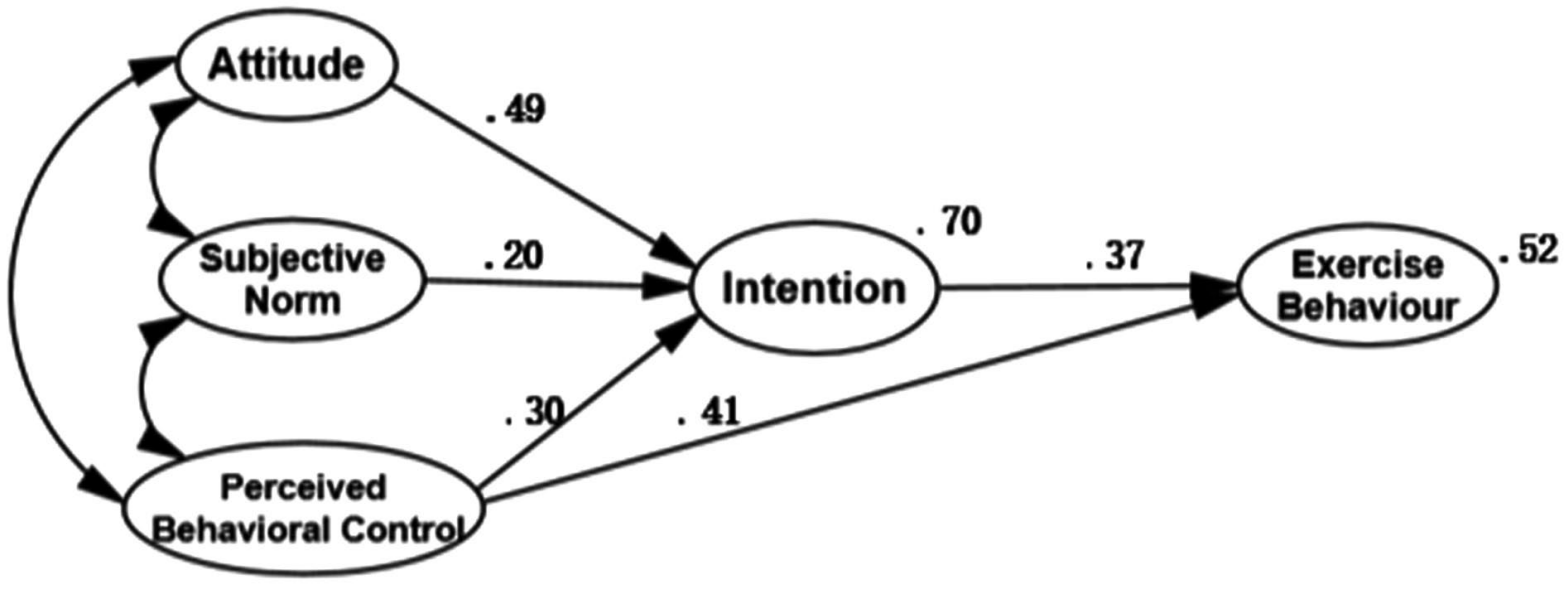
Path coefficient diagram of structural equation model for TPB-5.

The SEM parameter estimates are shown in Figure 2. The 5-factor TPB model had satisfactory model fit, *χ*^2^/*df* = 3.853 (*p* < 0.05), TLI = 0.930, CFI = 0.944, IFI = 0.944, GFI = 0.920, and RMSEA = 0.070. the path coefficients were significant and in the right direction for Attidude–Intention (standardized coefficient *β* = 0.49), Subjective norm–Intention (*β* = 0.20), and the Perceived behavioral control–Intention relationship (*β* = 0.30), Perceived behavioral control–Exercise behavior (*β* = 0.41), Intention–Exercise behavior (*β* = 0.37), all path coefficients are significant (*p* < 0.001). The SEM model explained 70% of the variance in intentions (*R*^2^ = 0.70) and 52% of the variance in exercise behavior (*R*^2^ = 0.52). The results suggested that the 5-factor TPB model is appropriate for predicting college students’ exercise behavior.

### Mediation and Moderation Effect of Exercise Commitment

We then tested the mediation and moderation effect of exercise commitment in the relationship between intentions and exercise behavior. We adopted the bias-correction bootstrap method (Hayes, 2013) with 5,000 re-samplings and 95% confidence interval (95% CI) using PROCESS macro to test both mediation and moderation effects. If the 95% CI do not include zero, it indicates significant effects.

#### Regression Models for Mediation Effect

Table 2 shows the regression model results for mediation effect testing purpose. After controlling for other variables in the TPB model, intentions positively and significantly predicted exercise behavior (*β* = 0.351, *p* < 0.001) and exercise commitment (*β* = 0.331, *p* < 0.001) in separate regression models. Intentions (*β* = 0.286, *p* < 0.001) and exercise commitment (*β* = 0.257, *p* < 0.001) jointly predicted exercise behavior.

**Table 2:** Regression analyses for testing the mediation effect of exercise commitment.

Regression models		Fit index			Regression parameters	
Outcome	Predictor	*R*	*R* ^2^	F	*β*	*t*
Exercise behavior	Intentions	0.526	0.276	220.917	0.351	14.863^***^
Exercise commitment	Intentions	0.529	0.28	225.185	0.331	15.006^***^
Exercise behavior	Exercise commitment	0.572	0.328	140.742	0.286	6.642^***^
	Intentions				0.257	9.5582^***^

*** *p* < 0.001.

As shown in Table 3, the indirect effect had bootstrapped 95% CI of [0.061, 0.130], which does not include zero, indicating significant mediation effect of exercise commitment in the relationship between intentions and exercise behavior. Further, the direct effect from intentions to exercise behavior was also significant [bootstrapped 95% CI was (0.203, 0.312)], suggesting that exercise commitment played a partial mediation role. The indirect effect accounted for 26.89% of the total effect from intentions to exercise behavior. Therefore, the results supported our research hypothesis.

**Table 3:** Results of bootstrapped 95% CI for mediation effect.

	Effect	Boot	95% CI		Variance explained (%)
		SE	Lower	Upper	
Direct effect	0.257	0.028	0.203	0.312	73.11
Indirect effect	0.095	0.018	0.061	0.13	26.89
Total effect	0.351	0.023	0.307	0.396	

#### Moderation Effect Testing Using Bootstrap Method

The moderation effect of exercise commitment in the relation between intentions and exercise behavior was tested using bias-corrected Bootstrap method with 5,000 re-samplings and 95% CI. PROCESS macro was used. Results showed that the moderation effect was not significant (*β* = −0.022, *t* = −0.714, *p >* 0.05; 95% CI = −0.080, 0.038), therefore, exercise commitment did not play a moderation role in the relation between intentions and exercise behavior.

The regression models showed that the inclusion of exercise commitment enhanced the prediction power of intentions to exercise behavior, and exercise commitment played a role of partial mediator in the relationship. We next tested the mediation effect using SEM technique to reveal a more comprehensive picture of the relationship among the variables.

### Prediction of Exercise Behavior Using the Extended TPB

**Figure 3:**
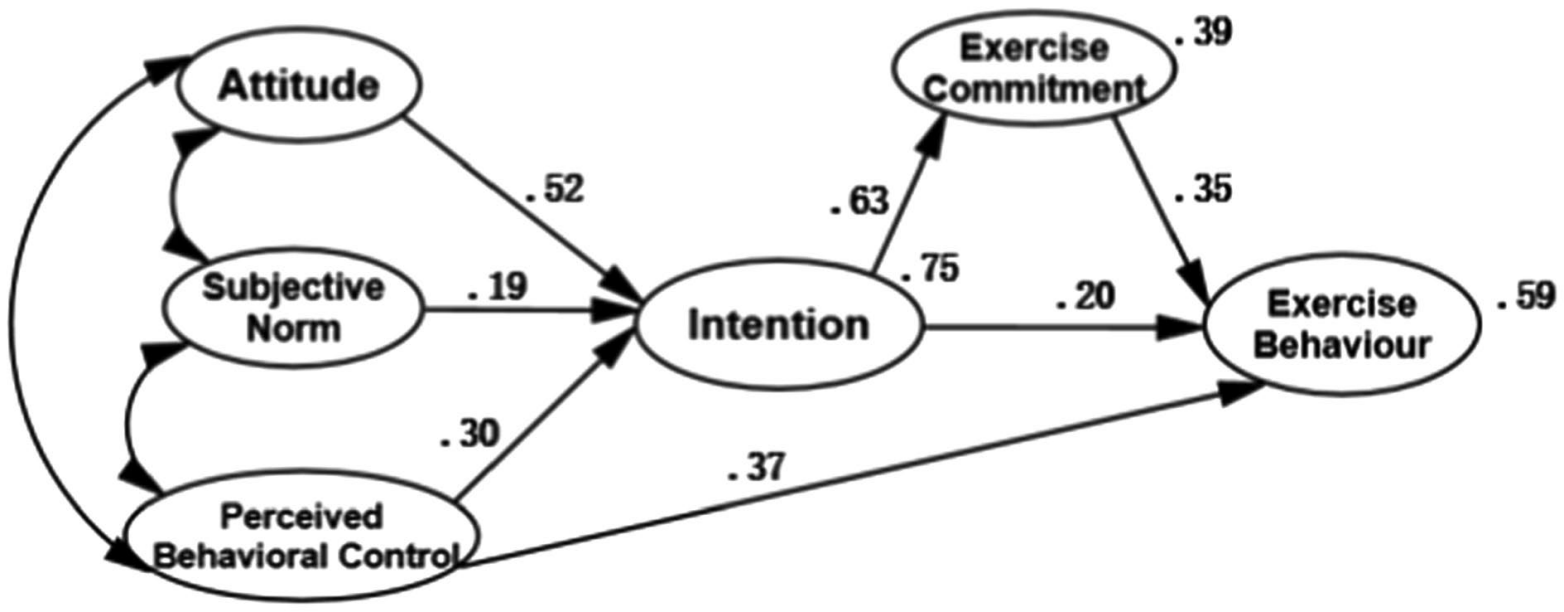
Path coefficient diagram of structural equation model for TPB-6.

Figure 3 shows the SEM model of the extended-TPB model predicting exercise behavior. The model had satisfactory fit, *χ*2/*df* = 3.948 (*p* < 0.05), TLI = 0.906, CFI = 0.918, IFI = 0.919, and RMSEA = 0.071. The SEM model explained 75% variance in intentions (*R*^2^ = 0.75), and 59% variance in exercise behavior (*R*^2^ = 0.59). Compared to the 5-factor TPB model, the 6-factor TPB model explained 7% more variance in exercise behavior.

As shown in Figure 3, attitude, subjective norms, and perceived behavioral control significantly predicted exercise intentions, and exercise intentions significantly predicted exercise commitment and behavior. Moreover, exercise intentions, commitment, and perceived behavioral control significantly predicted exercise behavior. With the inclusion of exercise commitment, the prediction power towards exercise behavior increased 7%.

Our results showed that exercise commitment played the role of a partial mediator in the relation between exercise intentions and behavior, and significantly enhanced the prediction power of TPB model to exercise behavior. Therefore, the extended 6-factor TPB model can be used to predict exercise behavior among college students. These results supported our research hypothesis.

## Discussion

### Analysis of Applicability of the 5-Factor Model of Physical Exercise

The conclusion of this study supports the notion of TPB that exercise intention is the strongest predictive variable of exercise behavior (Rivis and Sheeran, 2003; Ajzen and Fishbein, 2005; Beville et al., 2013; Zhang and Mao, 2016; Yang et al., 2020). The results revealed that the college students’ exercise intention mainly depends on their attitude towards exercise, subjective norms and perceived behavioral control, among which attitude and perceived behavioral control account for the largest proportion of variance. Therefore, when promoting college students’ participation in physical activity and implementing interventions, it is important to enhance their positive cognitive level and self-control of physical activity. When college students have positive attitudes toward exercise and perceive more favorable factors and fewer hindering factors in the process of implementing exercise behaviors, this will lead to stronger exercise intentions and behaviors. Indicating that college students’ positive attitude towards physical exercise and their sense of behavioral control and efficacy in exercise execution are more important for the formation of behavioral intention and the execution of final behavior, the results are consistent with that of Xu et al. (2018) and Wu (2020) study. The subjective norms of college students are injunctive norms, and they have a high-level self-determination and autonomy, so the pressure of injunctive norms from significant others has less effect on college students’ intention to exercise. As a result, their perceived subjective norms are lower. The results also show that behavioral intention and perceived behavioral control are both effective predictors of exercise behavior, and both have remarkable explanatory power for exercise behavior, among which behavioral intention is more helpful to exercise behavior. The results of the study support the hypothesis 1.

### Mechanism of Exercise Commitment on the Transformation From Exercise Intention to Behavior

Mediation and moderation effect of exercise commitment was tested with the bootstrapped confidence interval. Results showed that exercise commitment has a partial mediating effect between exercise intention and behavior, which accounts for 26.89% of the total effect, and the moderating effect is less significant; the results are consistent with that of Dong (2013) and Kang et al. (2020) study. The results suggest that exercise intention may be transformed into behavior through exercise commitment, and it is meaningful for the prediction of exercise behavior to incorporate exercise commitment, in the promotion of exercise behavior, it is necessary to enhance college students’ attachment and loyalty to physical exercise and raise the level of exercise commitment. The results of the study support hypothesis 2.

The results verified that the 5-factor model of physical exercise can better predict exercise behavior of college students, and further verify that the model can improve the predictive power of exercise behavior after the introduction of exercise commitment. The results of the 5-factor model suggest that the total explained variance in intention is 70% and the explained variance in exercise behavior is 52%. In contrast, the explained variance in intention of 6-factor model with exercise commitment reaches 75%, which is 5% higher than the original model, and explained variance in exercise behavior is 59%, with an increase of 7%. The hypothesized 6-factor model has sound model fit and greater prediction power, and serves as a suitable predictive intervention model for college students; the results prove that in addition to higher exercise intention, college students should also have positive exercise perceptions and experiences, so as to enrich their exercise consciousness and cognitive system, equip the individuals who originally have higher exercise intentions with stronger exercise desire and determination, and then establish a continuously stable, regular and orderly exercise behavior, the results of the study support the hypothesis 3.

According to Beatty’s commitment model theory, commitment, internalized by emotional dependence, behavioral engagement and effect expectations, is the bridge between individuals’ cognition and behavior (Beatty et al., 1988). On the basis of this theory, the study confirms the significant mediating effect of exercise commitment between exercise intention and behavior. Exercise intention is the motivational basis for college students to participate in physical exercise, and exercise commitment is a high-level intrinsic motivation and contractual attitude to achieve the exercise motivation intensity and produce exercise behavior. It also reflects the loyalty of actual participation in exercise behavior, reflecting the motivation intensity and persistence of the process from individual exercise intention to behavior, thereby ensuring the transformation from behavioral intention to behavior, indeed, exercise commitment plays a mediating role between exercise intention and behavior. In conclusion, college students’ intention to participate in physical exercise can directly predict their behavior, and it can also act on behavior through the mediating effect of exercise commitment, but the direct effect of intention on behavior is stronger than the mediating effect of exercise commitment. The results imply that behavior execution is not only determined by individuals’ intention, but also influenced by other factors. The new variables added previously are single-sided and uncomprehensive explanatory behavior (e.g., social support, emotion, persistence, etc.), whereas exercise commitment includes such aspects as exercise enjoyment, personal investment, social constraint, participation opportunity and choice, which promotes exercise behavior more comprehensively and improves the explanatory power of behavior. Exercise commitment, as a rational psychological decision, stimulates individuals’ desire and determination to participate in physical exercise and provides strong motivation and behavioral volition for college students to engage in exercise activities. It is a bridge between individuals’ cognitive intention and behavior, and also a strong evidence for the formation of stable, active, regular, and orderly exercise. Individuals with strong exercise commitment usually have clear exercise goals and intentions, positive exercise experiences, as well as strong desire and determination to participate in physical exercise, who are more willing to devote themselves to physical exercise.

Social psychologists generally believe that behavioral commitment reflects the persistence, stability or continuity of individuals’ behavioral process (Qiu and Xu, 2018). Studies prove that exercise commitment is a comprehensive manifestation of individuals’ cognitive evaluation, emotional experiences and behavioral tendencies of exercise behavior, and internal efficacy of college students’ persistence in physical exercise, individuals’ desire and determination to participate in physical exercise will increase the possibility of actual participation. The results of this study also shows that the higher the commitment level of participation in physical exercise is, the better the exercise behavior will be, college students can devote themselves to physical exercise and be self-disciplined, and they have a sense of exercise efficacy and experience the joy of exercise. Providing opportunities and options for participation and strengthening social supports will have a positive impact on promoting physical exercise, that is, individuals with a high-level commitment to exercise behavior will participate in and maintain physical exercise more actively. Therefore, increasing the level of behavioral commitment and enhancing the attachment and inclination to exercise behavior is a positive and effective way to promote college students to participate in physical exercise.

By organizing various and appropriate forms of physical exercise, universities could provide more opportunities for students to participate in physical exercises, enrich the mode of exercises, stimulating college students’ enthusiasm and participation in exercises. The more positive emotional experience obtained through physical exercises, the more likely that college students would engage in physical exercises. At the same time, it would give students more space and opportunities to pursue autonomy, and reduce social and external constraints and pressure. Making physical exercise mandatory will cause college students to be more resistant to physical exercise. The high-quality environmental support from schools, sufficient sports exercise facilities, and appropriate layout of activity space help to enhance college students’ perceived sports value, motivation in sports participation, and hence promoting physical exercise behavior. College students who form positive and stable exercise intention and exercise commitment would autonomously engage in exercise activities under various conditions. The autonomy and enjoyment they obtain from doing physical exercises would facilitate their self-growth and the sustainable participation in physical exercises. Therefore, college students should participate in physical exercise with a positive attitude, integrate into sports activities, stimulate individual subjective initiative, actively participate in peer interaction, and reduce psychological stress reactions. Boycott of online games and reducing sedentary behavior, making exercise plans and publicly committing to complete them, setting up reward and punishment mechanisms, punching reminders through sports bracelets or sports fitness APPs, and reminding to exercise by ringing when they are sedentary, and sports fitness APPs help users record sports fitness indices, guide sports learning, and lead healthy lifestyles and social elements, which help overcome exercise laziness and strengthen exercise Experience. Find exercise partners to motivate and support each other together, play the positive role of peer leadership, enhance the fun of exercise, experience the sense of joy of exercise, mold physique, show personality, promote the interpersonal skills of college students, integrate physical exercise into life, constantly enrich their spiritual world, and achieve more effective and lasting exercise behavior promotion through different boosting strategies.

An important way to promote physical exercise among college students is to raise their level of exercise commitment through certain educational methods. For example, we create diversified physical education platforms such as sports clubs, sports teams, sports festivals, sports APPs, digital sports smart devices, etc. We regularly send information on health knowledge and physical exercise skills through the school’s WeChat public number to facilitate students’ reading and learning at any time, and provide online support for students’ physical exercise. Record running clock and fitness through the WeChat sports APP and Keep APP, students are regularly selected as “exercise star” and the campus marathon and fitness exercise star game to stimulate positive imitation behavior and exercise motivation through the role model effect and group motivation. Strengthen the intelligent management and resource utilization rate of sports venues to fully meet the space needs of college students for physical exercise. Strengthen the construction of campus sports culture, build a social model of students’ sports exercise through the social collaboration function of sports clubs and sports festivals, expand the attractiveness and cohesiveness of “campus sports circle,” and motivate students to participate in leisure sports exercise after class. It is also required that students in grades 1–2 elective physical education courses to master sports skills and cultivate sports interests, while combining digital sports punch cards with sports exams, conducting regular physical health tests for college students, providing intervention programs through accurate analysis of physical health data, and establishing scientific and reasonable sports practice methods with the core of improving college students’ physical quality. Therefore, school physical education should focus on stimulating students’ motivation for physical exercise, creating an atmosphere to promote physical exercise and positive effect perception of exercise, helping students to realize and experience the benefits of physical exercise, such as and, entertainment, social networking, and skill development. By promoting students’ psychological inclination and attachment to participate in physical exercise and their attachment to exercise itself, college students are likely to consciously and actively participate in physical exercise, develop good exercise habits and then achieve the goal of lifelong physical exercise.

In response to the high intention–low behavior in the exercise model, the stronger the individual’s commitment and determination to participate in physical exercise, the more likely they are to make sustained effort to achieve the exercise goal, and the more positive, conscious, and enthusiastic their performance will be in physical exercise. Therefore, to promote college students’ physical exercise behaviors, the formation, maintenance and enhancement of exercise commitment should be promoted in certain ways to bring into play the positive effects of exercise commitment. In this regard, future research should focus on the interventions of exercise commitment and provide a set of effective intervention methods to promote physical exercise behavior of college students.

## Conclusion and Recommendations

### Conclusion

(1) The 5-factor model of physical exercise can better predict exercise intention and behavior of college students. (2) The 6-factor model of enhanced physical exercise is better than the 5-factor model in the prediction of intention and behavior, which is an applicable model for predictive interventions on college students’ physical exercise behavior. (3) Exercise commitment plays a partial mediating role between exercise intention and behavior, and it has no moderating effect.

### Recommendations

This study is cross-sectional, and the data are collected through self-reported surveys, making it difficult to grasp the dynamic characteristics of exercise commitment. It is recommended that future studies should conduct longitudinal experimental intervention studies, so as to explain the causal effect more effectively. In order to reduce the common method bias, more objective measuring tools (e.g., pedometer, accelerometer, and heart rate monitor) should be used to measure the actual exercise behavior of college students. TPB is not an exhaustive model, and there is plenty of room left for other mediators, moderators and predictors beyond the basic model. Future studies need to further integrate other social cognitive variables to enhance the predictive power of the model, and provide theoretical guidance and promotion strategies for the overall development of physical exercise and fitness of college students.

## Data Availability Statement

The original contributions presented in the study are included in the article/supplementary material, further inquiries can be directed to the corresponding author.

## Ethics Statement

The studies involving human participants were reviewed and approved by the Ethics Committee of Qufu Normal University. The participants provided their written informed consent to participate in this study.

## Author Contributions

W-JZ and Z-XM conceived and designed the study. W-JZ and Y-JF drafted the manuscript. Z-YY and T-FF collected the data and controlled the quality. Z-XM and MX revised the manuscript. W-JZ conducted the data analyses. All authors contributed to the article and approved the submitted version.

## Conflict of Interest

The authors declare that the research was conducted in the absence of any commercial or financial relationships that could be construed as a potential conflict of interest.

## Publisher’s Note

All claims expressed in this article are solely those of the authors and do not necessarily represent those of their affiliated organizations, or those of the publisher, the editors and the reviewers. Any product that may be evaluated in this article, or claim that may be made by its manufacturer, is not guaranteed or endorsed by the publisher.
